# Minimalinvasiver Tibialis-anterior-Sehnentransfer in Shuttle-Technik zur Behandlung des Klumpfußrezidivs im Wachstumsalter

**DOI:** 10.1007/s00064-022-00776-5

**Published:** 2022-07-04

**Authors:** Milena M. Ploeger, Matthias D. Wimmer, Charlotte Rommelspacher, Rahel Bornemann, Richard Placzek

**Affiliations:** Klinik für Orthopädie und Unfallchirurgie, Universitätsklinikum Bonn, Venusberg-Campus 1, 53127 Bonn, Deutschland

**Keywords:** Minimal-invasive OP Technik, Vereinfachte Nachbehandlung, Tibialis-anterior-Sehnentransfer, Klumpfußrezidiv, Klumpfuß, Minimal-invasive surgical technique, Simplified postoperative treatment, Tibialis anterior tendon transfer, Clubfoot recurrence, Clubfoot

## Abstract

**Operationsziel:**

Der Tibialis-anterior-Transfer in Shuttle-Technik mittels Tunnelator ist eine atraumatische Operationstechnik ohne Verletzung des Retinaculum extensorum. Im Vergleich zur aktuellen Literatur ermöglicht die Technik eine vereinfachte postoperative Nachbehandlung mit schmerzadaptierter Vollbelastung für 6 Wochen im Unterschenkelgehgips.

**Indikationen:**

Passive korrigierbare Klumpfußrezidive bei muskulärer Dysbalance (überbalancierter Tonus des M. tibialis anterior).

**Kontraindikationen:**

Strukturell bedingte Bewegungseinschränkungen des Fußes, muskuläre Insuffizienz des M. tibialis anterior, infektiöses oder tumoröses Geschehen im Operationsgebiet.

**Operationstechnik:**

Lösen des M. tibialis anterior an der Basis des Os metatarsale I. Führen der Sehne nach proximal aus dem Retinaculum extensorum, anschließendes Shutteln der Sehne unterhalb des Retinaculum mithilfe des „Tunnelators“ und transossäre Fixierung am Os cuneiforme laterale.

**Weiterbehandlung:**

Schmerzadaptierte Vollbelastung im Unterschenkelgehgips für 6 Wochen postoperativ.

**Ergebnisse:**

Im Zuge einer retrospektiven Studie wurde zwischen 2013 und 2019 das oben genannte operative Verfahren bei 20 Patienten (insgesamt *n* = 26 Operationen) durchgeführt. Nach einem Follow-up von 12 Monaten zeigte sich in 88,5 % der nachuntersuchten Fälle eine vollständige Korrektur des Klumpfußrezidivs. Es kam zu keinen allgemeinen oder spezifischen Operationskomplikationen.

## Abstract (en)

**Objective:**

Tibialis anterior transfer in shuttle technique using the tunnelator is an atraumatic surgical technique without injuring the extensor retinaculum. The technique allows for postoperative treatment with pain-adapted full weight-bearing for 6 weeks in a lower leg cast which is simplified compared to the current literature.

**Indications:**

Passive, correctable clubfoot recurrences in the event of muscular imbalance (overbalanced anterior tibial muscle).

**Contraindications:**

Structural movement restrictions of the foot, muscular insufficiency of the anterior tibial muscle, infection or cancerous lesions in the surgical site.

**Surgical technique:**

Loosen the tibialis anterior muscle at the base of the first metatarsal. Guide the tendon proximally out of the extensor retinaculum, then shuttle the tendon below the retinaculum with the help of the “tunnelator” and transosseous fixation at the lateral cuneiform.

**Postoperative management:**

Pain-adapted full weight-bearing in a lower leg walking cast for 6 weeks postoperatively.

**Results:**

In the course of a retrospective study, the above-mentioned surgical procedure was carried out in 20 patients (total *n* = 26 surgeries) between 2013 and 2019. After a follow-up of 12 months, complete correction of the clubfoot relapse was found in 88.5% of cases. There were no general or specific surgical complications.

## Vorbemerkungen

Die Behandlung des kongenitalen Klumpfußes erfolgt üblicherweise im Säuglingsalter nach der Ponseti-Methode [2]. Das Ziel der Ponseti-Redression ist eine subtalare Derotation durch Abduktion des Vorfußes nach Anhebung des ersten Strahls zur Korrektur des Hohlfußes. Neben der Gipsredression gehört zum Ponseti-Schema als weichteiliger Eingriff die perkutane Achillotenotomie ab einer Vorfußabduktion von 70° zur Korrektur der Spitzfußkomponente [8]. Die Rezidivrate nach Behandlung mit dem Ponseti-Schema wird in der Literatur recht heterogen angegeben und schwankt zwischen 20 und 41 % [1, 6].

Als erstes Anzeichen eines Klumpfußrezidivs zeigen sich in der klinischen Untersuchung häufig die Achillessehnenverkürzung sowie der Rückfußvarus. In der Schwungphase kommt es zu einer vermehrten Supinations- und Adduktionshaltung des Fußes [11]. Unterschieden werden das frühe Rezidiv innerhalb der ersten 2 Lebensjahre, welches in der Regel mit einer erneuten Ponseti-Gipsredression und ggf. erneuter perkutaner Achillotenotomie behandelt werden kann, und das späte Rezidiv ab dem 3. Lebensjahr.

Zur Behandlung der späten Rezidivkomponente beschrieben erstmalig Garceau und später auch Ponseti als Teil des Ponseti-Behandlungsschemas den Tibialis-anterior-Sehnentransfer (TAST) [4, 13]. Hierbei erfolgt das Lösen der Tibialis-anterior-Sehne im Bereich der Basis des Os metatarsale 1 und wird auf das Os cuneiforme mediale oder laterale transferiert. Alternativ veröffentlichten Hoeffer et al. erstmals 1974 den Teiltransfer der Tibialis-anterior-Sehne bei Kindern mit Zerebralparese [9]. In der Arbeit von Ponseti und Smoley erfolgt die Transposition ohne Lösen der Sehne aus dem Retinaculum extensorum mit 2 Inzisionen im Bereich des Fußrückens [13]. Andere Autoren beschreiben eine dritte Inzision auf Höhe oder proximal des Retinaculum extensorum mit Herauslösen der Sehne aus dem Begleitgewebe und Neupositionierung [11]. Die Fixation erfolgt laut Ponseti in der Regel transossär mit anschließender Ruhigstellung im Unterschenkelgips [13].

Bis dato werden in der Literatur mehrere operative Modifikationen des TAST beschrieben. Unterschiede zeigen sich bezüglich der Neupositionierung der Sehne (Belassen im Retinaculum extensorum vs. Neupositionierung) sowie in der Fixationstechnik der Sehne (Fadenanker [16], Kirschner-Draht-Fixierung [18] oder Interferenzschrauben [17]). Die postoperative Behandlung erfordert eine 6‑wöchige Ruhigstellung im Unterschenkelgips mit Teil- bzw. Entlastung an Unterarmgehstützen [13, 18]. Bis dato zeigt sich keine klare Überlegenheit eines operativen Verfahrens [12]. In der Kadaverstudie von Knutsen et al. führt der totale Sehnentransfer mit 3 Inzisionen zu einer verbesserten Korrektur der Fehlstellung im Vergleich zum partiellen Sehnentransfer und dem totalen Sehnentransfer mit 2 Inzisionen [10].

Die Verfahren, die die Sehne nach proximal aus dem Retinaculum extensorum herauslösen, nutzen zur Neupositionierung eine Kornzange, welche unterhalb des Retinaculums hindurchgeschoben wird [3]. Bei sehr jungen Patienten ist diese Methode oft erschwert aufgrund der anatomischen Enge. Hierdurch kann es zu Verletzungen der umgebenden Strukturen kommen.

In der vorliegenden Publikation beschreiben wir erstmals den TAST mittels Tunnelierer als atraumatische Operationstechnik ohne Verletzung des Retinaculums. Im Vergleich zur aktuellen Literatur favorisieren wir eine vereinfachte postoperative Nachbehandlung mit schmerzadaptierter Vollbelastung für 6 Wochen im Unterschenkelgehgips, die gerade beim jungen Patientenkollektiv zu einer einfacheren Umsetzung und guten Compliance führt [5].

## Operationsprinzip und -ziel

Identifikation der M.-tibialis-anterior-Sehne über einen Schnitt proximal des Retinaculum extensorum im Übergang des mittleren zum distalen Drittel der Tibiavorderkante, anschließend Lösen des Sehnenansatzes möglichst knochennah im Bereich der Basis des Os metatarsale I. Führen der Sehne nach proximal aus dem Retinaculum extensorum, anschließendes Shutteln der Sehne unterhalb des Retinaculum mithilfe des „Tunnelators“ und transossäre Fixierung am Os cuneiforme laterale.

### Vorteile

- Atraumatisches Vorgehen durch Shutteln der Sehne mittels „Tunnelator“
- Keine präformierten Höhlen
- Geringeres Risiko der Einblutung z. B. bei Hämophilie
- Postoperative schmerzadaptierte Vollbelastung im Gehgips
- Verbesserte Dorsalextension bei komplettem Tibialis-anterior-Transfer [10]
- Verbesserte Neutralstellung
- Keine Fixierung plantar mit möglicher Affektion des N. plantaris

### Nachteile

- Mögliche Überkorrektur bei Verlagerung des Sehnenansatzes zu weit lateral
- Mögliche Unterkorrektur bei Verlagerung des Sehnenansatzes zu weit medial

### Indikationen

- Passive korrigierbare Klumpfußrezidive bei muskulärer Dysbalance (überbalancierter Tonus des M. tibialis anterior)

### Kontraindikationen

- Strukturell bedingte Bewegungseinschränkungen des Fußes
- Muskuläre Insuffizienz des M. tibialis anterior
- Lokale Hautläsionen im Operationsgebiet
- Lokale Infektionen im Operationsgebiet
- Allgemeine Kontraindikation, wie z. B.: systemische Infektionen, eingeschränkte Narkosefähigkeit

### Patientenaufklärung

- Allgemeine Operationsrisiken (Infektion, Thrombose, Embolie, Gefäß- oder Nervenschäden, Nachblutung, Wundheilungsstörungen u. a.)
- Unzureichende Korrektur mit konsekutiver muskulärer Dysbalance (Überkorrektur/Unterkorrektur)
- Ausriss an der Re-Insertionsstelle nach erfolgtem Sehnentransfers
- Erneutes Klumpfußrezidiv
- Druckulzera/Nervendruckschädigung durch Gips
- Nervenschäden mit konsekutiver Dysästhesie, Kraftminderung
- Postoperative schmerzadaptierte Vollbelastung im Gehgips für 6 Wochen
- Unterschenkelgips für 6 Wochen postoperativ
- Stationärer Aufenthalt von ca. 2 Tagen postoperativ

## Operationsvorbereitungen

### Instrumentarium

- „Tunnelator“ (z. B. Peritoneal-Tunnelierer, Fa. Integra®, Saint Priest, Frankreich)
- 2er-Vicryl zur Armierung der Sehne
- Ledige Knochennadel zur ossären Transfixation
- Pfriem
- Zubehör zur Weißgipsanlage (Gewebeschlauch, Watte, Krepppapier, Weißgipsrollen, handwarmes Wasser)
- Bildverstärker mit Dosisreduktionseinstellung (mit Kupferfilter)

### Anästhesie und Lagerung

- Intubationsnarkose. Hilfreich ist eine Muskelrelaxation
- Durchleuchtbarer Operationstisch (z. B. Carbontisch)
- Rückenlagerung des Patienten
- Bewegliches Abdecken des zu operierenden Beines
- Keine Blutsperre, dadurch Vermeidung vielfacher assoziierter Risiken [15].

## Operationstechnik

**Figure 1:**
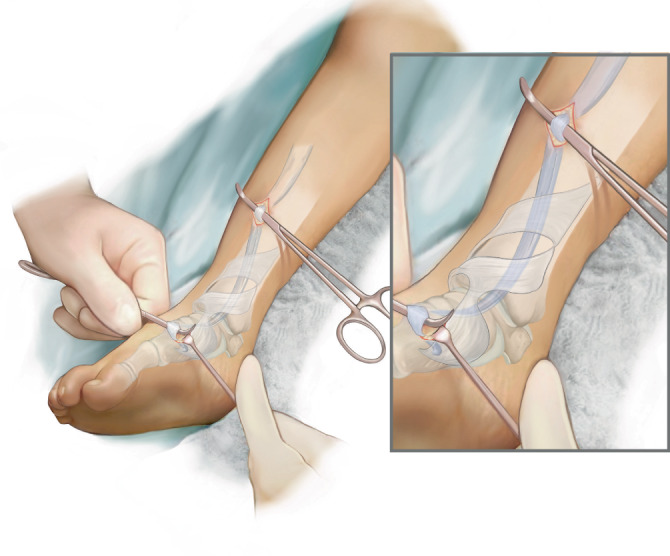

**Figure 2:**
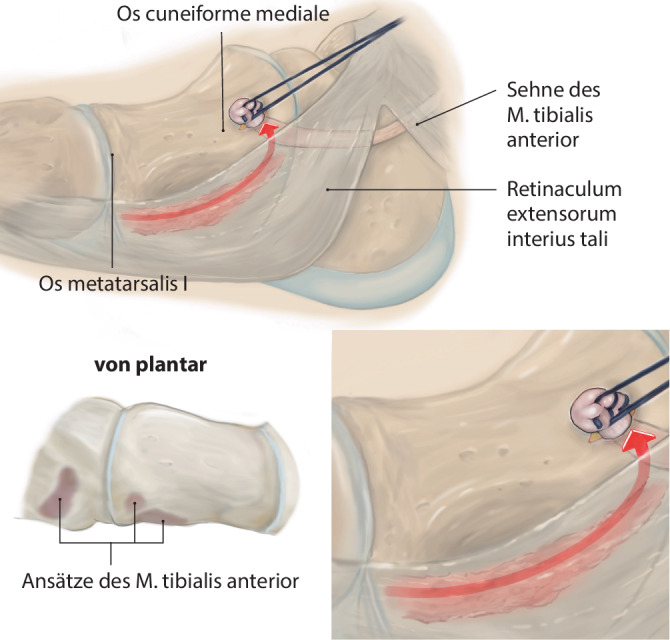

**Figure 3:**
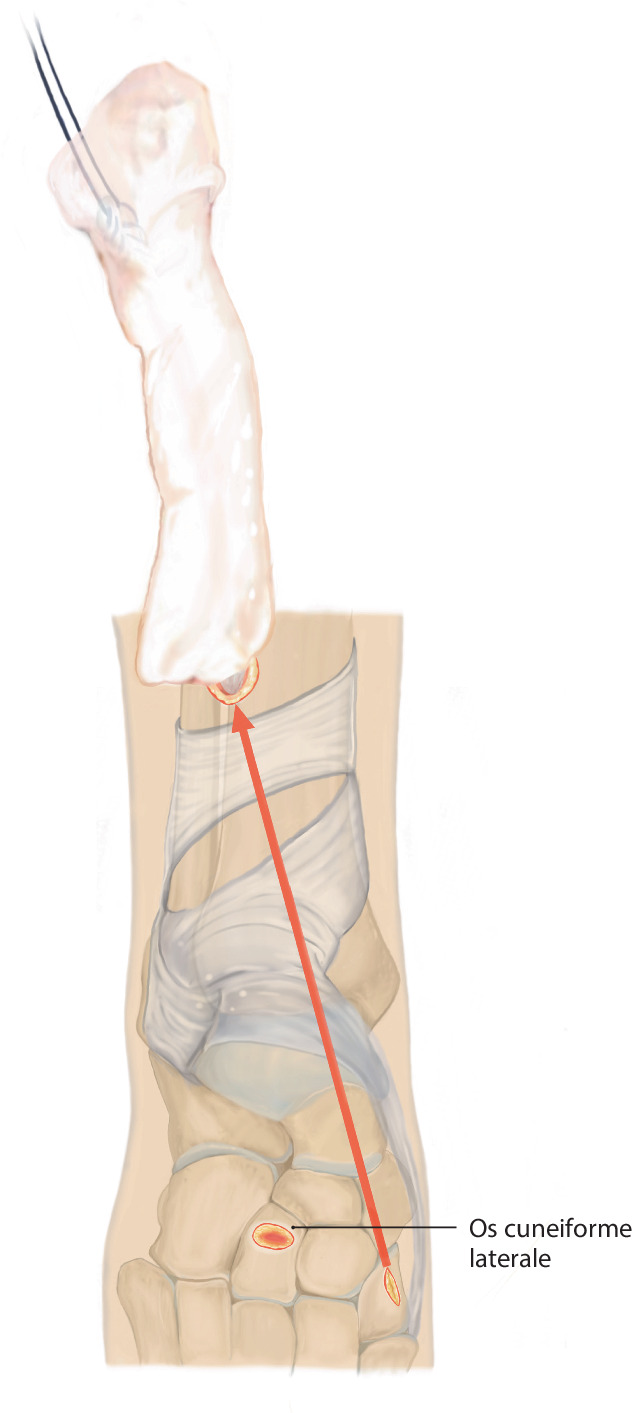

**Figure 4:**
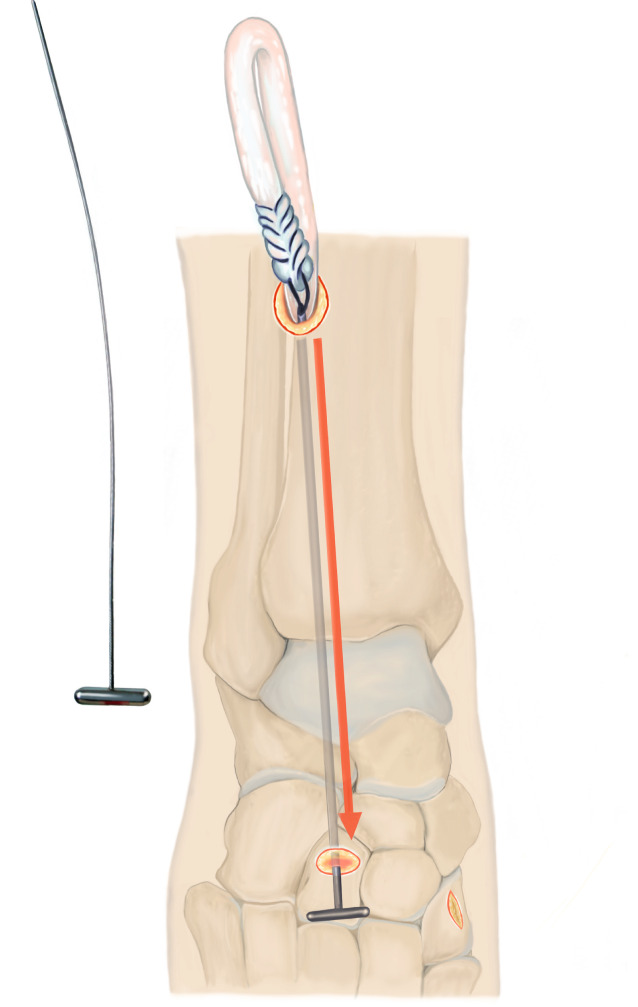

**Figure 5:**
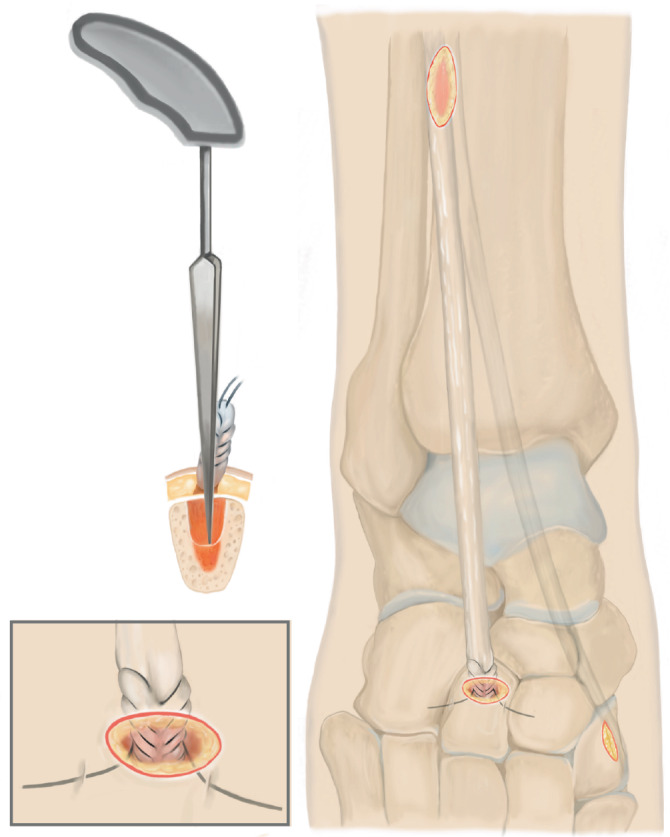

**Figure 6:**
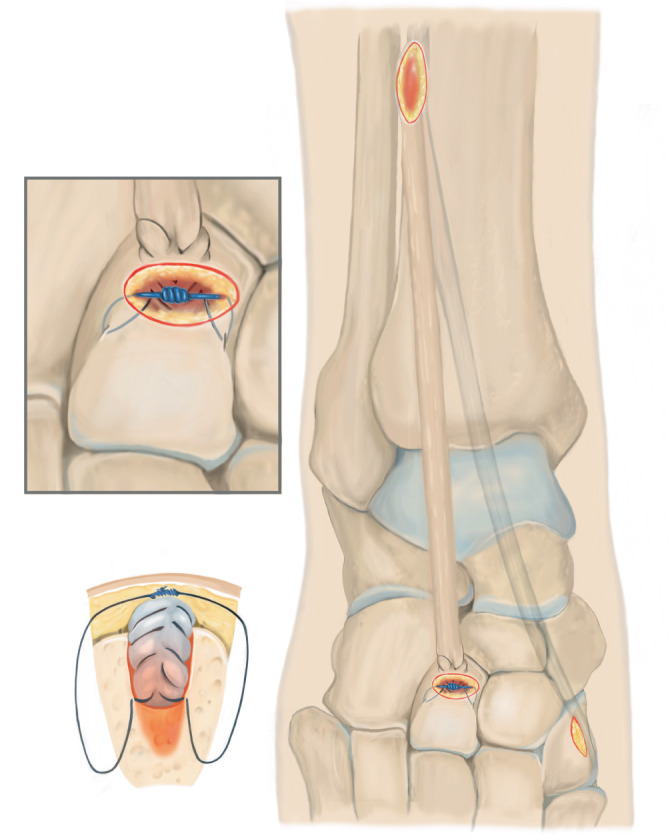

Abb. 1, 2, 3, 4, 5, 6, 7 und 8.

**Figure 7:**
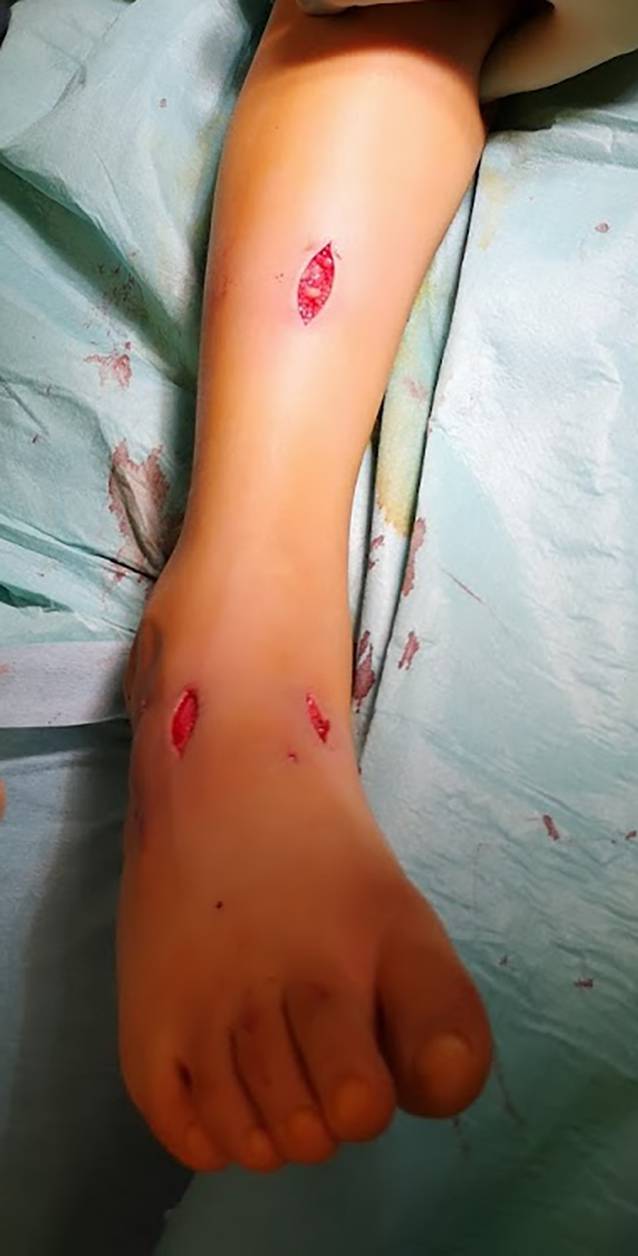

**Figure 8:**
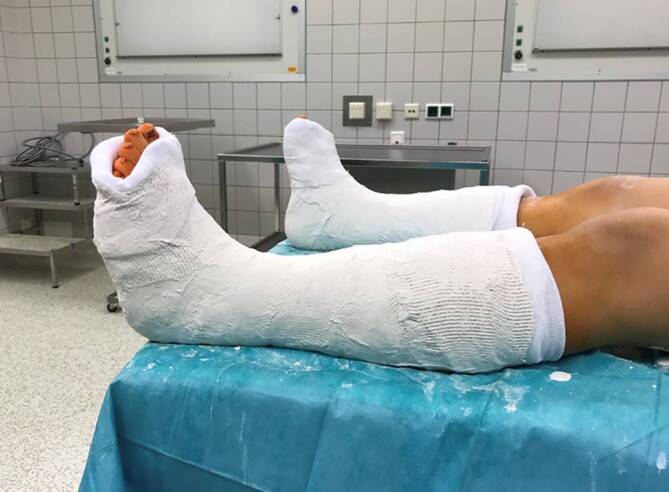

### Besonderheiten

- Erster Schnitt proximal des Retinaculum extensorum, sodass dieses nicht verletzt wird
- Atraumatisches Neupositionieren durch Shutteln der Sehne mithilfe des Tunnelators
- Kein plantares Fixieren/Ausstechen der Sehnennaht (Vermeidung von Wundheilungsstörungen plantar sowie Vermeidung eines N.-plantaris-lateralis-Entrapments) [14]
- Intraoperative Röntgenkontrolle in der Regel nicht notwendig
- Nach Hybridisierung des Weißgipses schmerzadaptierte Vollbelastung möglich

### Postoperative Behandlung

- Intraoperative zirkuläre Unterschenkelgipsanlage mit Weißgips in Neutralstellung
- Nach abschließendem Aushärten des Weißgipses Hybridisierung auf Gehgips mit synthetischem Stützverband. Die Autoren hybridisieren den Gips in der Regel am 1. postoperativen Tag
- Tragedauer des Gipses 6 Wochen postoperativ
- Vollbelastung im Gehgipses
- Gipsabnahme ambulant 6 Wochen postoperativ
- Sportkarenz für insgesamt 8 Wochen postoperativ, adaptiert an die individuellen Voraussetzungen

### Fehler, Gefahren, Komplikationen

- Nur partielles Lösen des Sehnenansatzes (Verbleibt der anteriore Anteil auf dem Os metatarsale I, ist das Zurückziehen der Sehne nach proximal nicht möglich)
- Zu weit proximales Ablösen des Sehnenansatzes kann dazu führen, dass der armierbare Sehnenanteil zur Refixation zu kurz ist (ein möglichst knochennahes Ablösen ist daher essenziell)
- Durchführen des Tunnelators unterhalb des Retinaculums sicherstellen
- Keine Manipulation mehr am Fuß nach transossärer Fixierung bis zur Gipsanlage, um ein Ausreißen der Sehne zu vermeiden. Anderes „Stabilitätsempfinden“ der Refixation als beim Erwachsenen Sehnen-Graft berücksichtigen!

## Ergebnisse

Im Zuge einer retrospektiven Studie wurde zwischen 2013 und 2019 das oben genannte operative Verfahren bei 20 Patienten (weiblich *n* = 7 [35 %], männlich *n* = 13 [65 %]) durchgeführt. Bei 6 Patienten erfolgte der TAST bilateral, sodass insgesamt *n* = 26 TAST nachuntersucht wurden. Das Durchschnittsalter zum Operationszeitpunkt lag bei 8 Jahren und 1 Monat (MIN = 3 Jahre, MAX = 38 Jahre). Das Follow-up betrug im Mittel 43,9 Monate (SD ± 24,9 Monate, MIN = 3 Monate, MAX = 78 Monate). Zwei Patienten wurden in das Follow-up nicht eingeschlossen, da nach Gipsabnahme (6 Wochen postoperativ) die weiteren Kontrollen heimatnah erfolgen.

Bei *n* = 17 Patienten bestand die Indikation im Rezidiv eines idiopathischen Klumpfußes, bei *n* = 3 Patienten war die ursächliche Erkrankung ein neurogener Klumpfuß.

Als zusätzliche Operationen an Fuß und Sprunggelenk erfolgte bei 90 % der Patienten eine perkutane Achillotenotomie nach Hoke [7]. Weitere zusätzliche Operationen waren das perkutane Plantarfaszienrelease (*n* = 9 Patienten). Bei 11,5 % der operierten Fälle (*n* = 3 Füße) zeigte sich ein Rezidiv nach 12 Monaten. Bei 2 der 3 Patienten zeigt sich im Rahmen der Follow-up-Untersuchung nach 12 Monaten eine erneute Achillessehnenverkürzung, welche in 1 Fall erneut operativ behandelt wurde mittels perkutaner Achillotenotomie, im zweiten Fall erfolgte eine intensive physiotherapeutische Behandlung mit Aufdehnen der Achillessehne. Der dritte Patient hatte zudem neben der Achillessehnenverkürzung eine erneute Hohlfußkomponente, beides wurde operativ mit einer perkutanen Achillotenotomie und einem perkutanen Release der Plantarfaszie behandelt.

Bei der beschriebenen Fallserie ist es zu keinen allgemeinen oder neben den beschriebenen Rezidivfällen spezifischen Operationskomplikationen gekommen. Insbesondere die spezifischen technischen Risiken wie Sehnenausriss oder Über‑/Unterkorrektur traten nicht auf.
